# Survival prediction in triple-negative breast cancer: a Cox model with fairness assessment using ISO/IEC TR 24027:2021 in a MENA cohort

**DOI:** 10.37349/etat.2026.1002362

**Published:** 2026-03-20

**Authors:** Mehrshad Alirezaei Farahani, Fateme Sadeghipour, Hamid Reza Marateb, Maryam Soltan, Azar Naimi, Marjan Mansourian

**Affiliations:** University of Toronto, Canada; ^1^Biomedical Engineering Department, Engineering Faculty, University of Isfahan, Isfahan 81746-73441, Iran; ^2^Institute for Research and Innovation in Health (IRIS), Automatic Control (ESAII), Universitat Politècnica de Catalunya-BarcelonaTech (UPC), 08028 Barcelona, Spain; ^3^Center for Innovation and Development of Artificial Intelligence Technologies, University of Isfahan, Isfahan 81746-73441, Iran; ^4^Department of Pathology, School of Medicine, Isfahan University of Medical Sciences, Isfahan 81746-73461, Iran; ^5^Epidemiology and Biostatistics Department, School of Health, Isfahan University of Medical Sciences, Isfahan 81746-73461, Iran

**Keywords:** triple-negative breast cancer (TNBC), survival prediction, Cox proportional hazards model, fairness in AI, ISO/IEC TR 24027:2021, clinical decision support

## Abstract

**Aim:**

Triple-negative breast cancer (TNBC) is an aggressive subtype with limited therapeutic options and poor survival outcomes. Prognostic models developed in Western cohorts rarely assess algorithmic fairness. This study aimed to develop and internally validate a clinically interpretable Cox survival model for TNBC using baseline diagnostic variables and to evaluate its fairness according to ISO/IEC TR 24027:2021 guidelines in a Middle East and North Africa (MENA) cohort.

**Methods:**

A total of 138 TNBC patients were included after merging two institutional datasets and removing variables with > 25% missingness. Baseline features comprised age, tumor size, lymph node involvement, tumor grade, Ki-67, type of surgery, metastasis at diagnosis, chemotherapy, and radiotherapy. A Cox proportional hazards (CoxPH) model with six clinically established predictors was fitted to reduce overfitting. Model performance was assessed through five-fold stratified cross-validation using Harrell’s concordance index (C-index), receiver operating characteristic area under the curve (AUROC), and calibration curves. Fairness was evaluated using demographic parity, equality of opportunity, predictive equality, and equalized odds metrics following ISO/IEC TR 24027:2021.

**Results:**

During follow-up, 34 patients (24.6%) died. Metastasis at diagnosis, high tumor grade, and radical mastectomy were significantly associated with mortality. The CoxPH model achieved a C-index of 0.80 [SE = 0.04; 95% confidence interval (CI): 0.72–0.87] and an AUROC of 0.81 (95% CI: 0.72–0.90). Calibration plots showed strong agreement between predicted and observed survival probabilities, with a modest overall bias of –8.8%. Fairness assessment revealed small but notable disparities in false-positive rates across age groups and surgical categories, while lymph node status and other variables showed no significant bias.

**Conclusions:**

This study presents a robust and fairness-aware survival prediction model for TNBC using routinely available clinical features. The model demonstrates strong discrimination, good calibration, and quantifiable fairness across patient subgroups, offering a clinically interpretable and ethically aligned tool to support TNBC risk stratification and decision-making in the MENA region.

## Introduction

Breast cancer is one of the most challenging diseases in the world. It was the most common cancer in women in 157 countries out of 185 countries in 2022. The number of women with breast cancer in 2022 was 2.3 million, and the number of deaths due to this disease was 670,000 [1].

Triple-negative breast cancer (TNBC) is an aggressive type of breast cancer. This type of cancer is very difficult to diagnose due to the negativity of estrogen receptor (ER), progesterone receptor (PR), and human epidermal growth factor receptor 2 (HER2) and has fewer treatment options. Unfortunately, it is estimated that of the worldwide breast cancer burden, approximately 170,000 cases are TNBC, accounting for about 10–20% of invasive breast cancers [2, 3].

According to global estimates, the burden of breast cancer varies across the world, depending on the human development index of each country. The 5-year survival rate in developed and high-income countries is over 90 percent, while in less developed countries such as India and South Africa, the figures are 66 and 40 percent, respectively [4].

The World Health Organization’s Global Breast Cancer Initiative (GBCI) aims to reduce global breast cancer mortality by 2.5% per year, particularly in developed countries, by strengthening and developing health systems [1].

The field of breast cancer research has seen significant advancements over the past decades, with numerous studies contributing to our understanding of survival prediction across different molecular subtypes. In a 2023 study, Zarean Shahraki et al. [5] analyzed 3,580 breast cancer patients treated from 1991 to 2021 at Shahid Beheshti University’s Cancer Research Center. This study incorporated a comprehensive range of variables, including demographic, clinical, and treatment-related features, using time-to-event deep learning-based models, specifically the N-net survival model. A focused analysis of 632 patients with TNBC identified tumor stage, age at diagnosis, and lymph node status as key predictors. The efficacy of the N-net model was validated through cross-validation, demonstrating survival rates of 84%, 74%, and 66% at 5, 10, and 15 years, respectively, for triple-negative patients. It underscores its potential in enhancing outcome prediction in breast cancer management. It sets the stage for future research to further refine these models and expand our understanding of survival outcomes in breast cancer patients.

In the 2023 study by Zhou and Chen [6], 33,654 TNBC patients from the SEER database were analyzed to investigate prognostic factors and survival outcomes. The study incorporated a comprehensive range of variables, including demographic, clinical, and treatment-related features, and utilized both univariable and multivariable Cox regression to identify prognostic factors. The results indicated that younger, white, married individuals with lower disease grades, lower TNM stages, and those who underwent surgery had better outcomes. Cancer-specific survival was notably better in patients who had surgery. The study included both internal and external validation, with the model’s efficacy validated through a hold-out method. The concordance index (C-index) for internal validation was 0.79, and for external validation, it was 0.79. Calibration plots for 3-year and 5-year survival were also provided, demonstrating the model’s accuracy.

In the 2023 study by Meng et al. [7], 32,836 non-metastatic TNBC patients from the SEER database, treated between 2010 and 2019, were analyzed to predict 10-year survival outcomes. The study incorporated a wide array of variables, including demographic, clinical, and treatment-related features, utilizing lasso regression. The model’s efficacy was validated using a hold-out method, with an area under the curve (AUC) ranging from 0.75 to 0.86. Additionally, the study included a calibration chart to demonstrate the model’s accuracy.

In the 2022 study by Chen et al. [8], 1,962 female patients with metastatic TNBC from the SEER database, treated between 2010 and 2017, were analyzed to determine breast cancer specific survival (BCSS) and overall survival (OS). The study incorporated a wide array of variables, including demographic and clinical features related to metastasis in different organs, as well as treatment-related features, and utilized Cox regression. The BCSS rates for 1-, 2-, and 3-year survival were 56.55%, 29.86%, and 19.71%, respectively. The OS rates for 1-, 2-, and 3-year survival were 52.55%, 26.03%, and 16.69%, respectively. The study included both internal and external validation. The model’s internal efficacy was validated using a hold-out method, and calibration curves for 1-, 2-, and 3-year BCSS were provided for both training and validation cohorts.

In the 2022 study by Sheng et al. [9], 636 TNBC patients treated between 2011 and 2015 were analyzed to predict disease free survival (DFS) models using a combination of clinical features, ultrasound, and mammography. The study incorporated various variables, including demographic, clinical, and treatment-related features, utilizing multivariate logistic regression and Cox regression. The model’s efficacy was validated using a holdout method. According to the nomogram prediction performance, the C-index for predicting DFS was 0.69 for the training cohort and 0.69 for the validation cohort. The AUCs for predicting 1-, 3-, and 5-year DFS were 0.78, 0.70, and 0.69 in the training cohort and 0.49, 0.72, and 0.69 in the validation cohort. Calibration curves showed excellent agreement between the survival probabilities predicted by the nomogram and the actual survival probabilities in both training and validation groups.

In the 2022 study by Huang et al. [10], 4,696 TNBC patients from the SEER database, treated between 2010 and 2016, were analyzed to assess the impact of chemotherapy on survival and predict OS. The study incorporated a wide array of variables, including demographic, clinical, and treatment-related features, utilizing nine machine learning models, with a focus on the LightGBM model. Cox regression was used to compare the survival of patients who received chemotherapy and those who did not, and the Kaplan-Meier test was used to generate survival curves. Log-rank tests identified differences in survival between patients who received chemotherapy and those who did not. The efficacy of the LightGBM model was validated through 10-fold cross-validation, achieving 85% accuracy and an AUC of 0.81 for OS.

In the 2021 study by Haiderali et al. [11], 450 TNBC patients from the USA were analyzed to predict event free survival (EFS), time to recurrence, and OS. Among these patients, 236 received neoadjuvant treatment, 72 received neoadjuvant treatment followed by surgery, 102 underwent surgery alone, and 40 did not receive any treatment or surgery. The study incorporated a wide array of variables, including demographic features and information about the initial diagnosis of the disease. Cox regression models were used to evaluate predictors of EFS and OS for within-group comparisons by pathological complete response (pCR) status. The mortality rates for the surgery, neoadjuvant, surgery with neoadjuvant, and no treatment groups were 23.6%, 16.5%, 14.7%, and 35%, respectively. The OS hazard ratios (HRs) were 1.04 for patients with high BMI, 0.27 for stage 2 cancer, and 0.2 for those with pCR.

In the 2021 study by Cui et al. [12], 4,593 TNBC patients aged 18 to 45 years, treated between 2010 and 2015 and included in the SEER database, were analyzed to investigate 3-year and 5-year OS and BCSS. Breast cancer-specific survival was defined as the interval from the time of diagnosis to the last follow-up or death from breast cancer. The study incorporated various variables, including demographic, clinical, and treatment-related features, utilizing a Cox regression model. The model’s efficacy was validated through a hold-out method. The AUC for 3-year OS was 0.78, for 5-year OS was 0.77, for 3-year breast cancer-specific survival was 0.78, and for 5-year breast cancer-specific survival was 0.77. The study also included a calibration chart demonstrating the model’s accuracy.

In the 2019 study by Shi et al. [13], 379 TNBC patients from China, treated between March 2008 and June 2014 at the First Affiliated Hospital of Wenzhou Medical University, were analyzed to predict the prognosis of TNBC. The model’s efficacy was validated through a hold-out method. In the validation group, the C-index for DFS was 0.69, and for OS was 0.74. The five-year receiver operating characteristic (ROC) curves were 0.66 for DFS and 0.70 for OS. Calibration graphs for 3- and 5-year OS and DFS were also provided, demonstrating the model’s accuracy.

In the 2019 study by Yang et al. [14], 296 invasive operable TNBC patients treated between 2002 and 2014 at Sun Yat-sen Memorial Hospital were analyzed to predict DFS and OS for operable TNBC based on Chinese breast cancer data. The study incorporated various variables, including demographic, clinical, and laboratory features, using Cox multivariate analysis. The efficacy of the multivariable Cox regression model was validated through bootstrapping. Additionally, 108 patients from the Second Xiangya Hospital and Peking University Shenzhen Hospital were considered for external validation. The C-index for DFS was 0.74 in the training group and 0.78 in the validation group. For OS, the C-index was 0.79 in the training group and 0.78 in the validation group.

In the 2018 study by Guo et al. [15], 21,419 TNBC patients treated between 2010 and 2015 from the SEER 19 Cancer Registry database were analyzed to predict BCSS and OS. The study incorporated various variables, including demographic and clinical features, utilizing log-rank tests, Cox analysis, and a competing risk model. The 1-, 3-, and 5-year probabilities of BC-specific mortality, as determined by hold-out validation, were 2.7%, 12.5%, and 17.1%, respectively. The study demonstrated internal and external validity, with C-index values of 0.78 for OS, 0.79 for BCSS in the internal validation, and 0.77 for OS and 0.792 for BCSS in the external validation. Calibration curves for internal and external validations were also provided to evaluate the accuracy of the survival nomogram.

In the 2018 study by Lin et al. [16], 404 TNBC patients treated between 2006 and 2012 at the Affiliated Union Hospital of Fujian Medical University were included in the training group, and 200 patients treated between 2012 and 2014 were included in the validation group to predict the prognosis of patients with TNBC, thus ensuring external validation. The study incorporated both clinical and laboratory features, utilizing univariate and multivariate Cox regression analyses. The C-index of the nomogram for predicting OS was 0.76 in the training cohort and 0.72 in the validation cohort. Calibration curves for three-year and five-year OS were also provided. Table 1 summarizes the state of the art.

**Table 1:** Summary of key studies on survival prediction in triple-negative breast cancer (TNBC), including cohort characteristics, feature sets, modeling approaches and performance metrics.

**Reference**	**Author, date**	**Outcome**	**Data specifications**	**Features**	**Method**	**Evaluation**	**Results**
[5]	Zarean Shahraki et al., 2023	Survival prediction in different molecular subgroups in TNBC	3,580 patients (1991–2021), Shahid Beheshti University’s Cancer Research Center, Iran	Demographic features such as age, clinical features such as tumor stage, and treatment-related features such as the type of surgery	Time-to-event deep learning-based models (N-net survival model)	Cross-validation	5-year survival probability risk: 84%, 10-year: 74%, 15-year: 66%
[6]	Zhou and Chen, 2023	Investigate prognostic factors and survival outcomes in TNBC	33,654 TNBC patients, SEER database, USA	Demographic features such as age, clinical features such as histologic grade, and treatment-related features such as surgery of the primary site	Univariable and multivariable Cox regression	Hold-out method, internal and external validation	C-index: 0.79 (internal), 0.79 (external) Better outcomes for younger, white, married individuals with lower disease grade and stage
[7]	Meng et al., 2023	Predict 10-year survival outcomes in non-metastatic TNBC patients	32,836 non-metastatic TNBC patients (2010–2019), SEER database, USA	Demographic features such as age at diagnosis, clinical features such as histologic grade, and treatment-related features such as surgery	The least absolute shrinkage and selection operator (Lasso) regression	Hold-out method	AUC: 0.86 for 1 year after diagnosis, 0.75 for 10 years after diagnosis
[8]	Chen et al., 2022	Determine BCSS and overall survival (OS) in female patients with metastatic TNBC	1,962 metastatic TNBC female patients (2010–2017), SEER database, USA	Demographic features such as age at diagnosis, clinical features such as tumor stage, and the feature of metastasis in different organs, such as bone metastasis	Cox regression	Hold-out method	1-year BCSS: 56.55%, 2-year: 29.86%, 3-year: 19.71% 1-year OS: 52.55%, 2-year: 26.03%, 3-year: 16.69%
[9]	Sheng et al., 2022	Predict disease free survival (DFS) models using a combination of clinical features, ultrasound, and mammography	636 TNBC patients (2011–2015), China	Demographic features such as age, clinical features such as histologic grade, and treatment-related features such as adjuvant chemotherapy	Multivariate logistic regression, Cox regression	Hold-out method, 0.69 (validation)	C-index: 0.69 (training), 0.69 (validation) 1-year DFS AUC: 0.78 (training), 0.49 (validation) 3-year DFS AUC: 0.70 (training), 0.72 (validation) 5-year DFS AUC: 0.69 (both training and validation)
[10]	Huang et al., 2022	Assess the impact of chemotherapy on survival and predict OS in TNBC	4,696 TNBC patients (2010–2016), SEER database, USA	Demographic features such as age, clinical features such as tumor status, and treatment-related features such as surgical approach	Nine ML models, with a focus on LightGBM	Ten-fold cross-validation	AUC: 0.81, accuracy: 85% for OS
[11]	Haiderali et al., 2021	Predict event free survival (EFS), time to recurrence, and OS in TNBC patients	450 TNBC patients (236 received neoadjuvant treatment, 72 received neoadjuvant therapy with surgery, 102 had only surgery, and 40 did not receive any treatment or surgery), USA	Demographics such as age at initial TNBC diagnosis, treatment-related features such as duration from initial TNBC diagnosis to surgery, and information about the initial diagnosis of the disease	Cox regression	Within-group comparisons by pCR status	Mortality rates: surgery (23.6%), neoadjuvant (16.5%), surgery + neoadjuvant (14.7%), no treatment (35%)
[12]	Cui et al., 2021	Investigate 3-year and 5-year OS and BCSS in TNBC patients	4,593 TNBC patients aged 18–45 (2010–2015), SEER database, USA	Demographic features such as age, clinical features such as tumor size, and treatment-related features such as breast surgery	Cox regression	Hold-out method	AUC: 0.78 (3-year OS) 0.77 (5-year OS) 0.78 (3-year BCSS) 0.77 (5-year BCSS)
[13]	Shi et al., 2019	Predict the prognosis of TNBC	379 TNBC patients from China (2008–2014), First Affiliated Hospital of Wenzhou Medical University, China	Demographic features such as age, clinical, and treatment-related features such as surgery type, blood factor features such as neutrophils	Univariate and multivariate Cox regression	Hold-out method	C-index: 0.69 (DFS) 0.740 (OS) 5-year ROC: 0.66 (DFS) 0.70 (OS)
[14]	Yang et al., 2019	Predict DFS and OS for operable TNBC based on Chinese breast cancer data	296 invasive operable TNBC patients (2002–2014), Sun Yat-sen Memorial Hospital (108 patients, The Second Xiangya Hospital and Peking University Shenzhen Hospital, external validation), China	Demographic features such as age, clinical features such as tumor size, and treatment-related features such as surgery type	Cox multivariate analysis	Bootstrapping, external validation	C-index: 0.74 (DFS training) 0.78 (DFS validation) 0.79 (OS training) 0.78 (OS validation)
[15]	Guo et al., 2018	Predict BCSS and OS in TNBC patients	21,419 TNBC patients (2010–2015), SEER 19 Cancer Registry, USA	Demographic features such as age, clinical features such as grade	Log-rank tests, Cox analysis, competing risk model	Hold-out validation	C-index: 0.78 (OS internal) 0.79 (BCSS internal) 0.77 (OS external) 0.79 (BCSS external) 1-year BC-specific mortality: 2.7%, 3-year: 12.5%, 5-year: 17.1%
[16]	Lin et al., 2018	Predict the prognosis of patients with TNBC	404 TNBC patients from the Affiliated Union Hospital of Fujian Medical University (2006–2012) for training, 200 patients (2012–2014) for validation, China	Clinical features such as tumor size, laboratory features such as CA125	Univariate and multivariate Cox regression	External validation	C-index of OS: 0.76 (training), 0.72 (validation)

AUC: area under the curve; C-index: concordance index; pCR: pathological complete response; ROC: receiver operating characteristic.

Despite substantial progress in TNBC prognostic modeling, several methodological gaps remain unresolved. Most existing models are derived from Western cohorts, leaving limited evidence for populations in the Middle East and North Africa (MENA), where patient characteristics and treatment patterns differ substantially. Additionally, although prior studies have applied diverse modeling approaches—including Cox regression, LASSO, and deep learning and have used large language models and transfer learning in health systems [17], few have conducted rigorous calibration assessments beyond visual plots, and almost none have quantified calibration bias or evaluated model reliability across risk strata. Crucially, no published TNBC survival model has incorporated algorithmic fairness assessment, and fairness frameworks such as ISO/IEC TR 24027:2021 remain entirely absent from the field. As a result, it is unknown whether existing models produce unequal error rates across clinically relevant subgroups such as age, surgical type, or nodal status. These gaps highlight the need for a region-specific, clinically interpretable model that integrates robust discrimination, rigorous calibration performance, and standardized fairness evaluation. Our study directly addresses these gaps using real-world TNBC data from the MENA region, and attempts to address the lack of transparency and unfair implementation despite the lack of data [18].

The remainder of this article is structured to follow the complete development, validation, and fairness assessment of our TNBC prognostic model. In the Materials and methods, we describe the cohort, preprocessing steps, variable definitions, and the construction of the Cox proportional hazards (CoxPH) model, followed by the fairness framework derived from ISO/IEC TR 24027:2021. The Results section presents descriptive characteristics of the cohort, model coefficients, discrimination measures (C-index and ROC-AUC), calibration performance, and fairness analyses across subgroups. In the Discussion, we interpret these findings in the context of prior literature, highlight the clinical implications of a fairness-aware survival model, and outline strengths and limitations. Finally, the article concludes with implications for clinical decision support in TNBC management within the MENA region and proposes directions for future bias-aware prognostic modeling research.

## Materials and methods

### Data collection and preprocessing

All cases were collected from the pathology archive database of Isfahan University of Medical Sciences. Medical records were reviewed to confirm the TNBC diagnosis from 2016 to 2021. Survival status information was obtained through review of medical records or telephone follow-up. Patients were included if they had confirmed TNBC (negative for ER, PR, and HER2). They excluded if they had died due to other causes unrelated to breast cancer or incomplete follow-up information. This study initially included two datasets. The first dataset consisted of 70 patients with the following variables: age, death status, time of death, Ki-67, presence of lymph node, history of comorbid conditions such as diabetes, cardiovascular disease, and hypertension, tumor size, menopausal status, type of surgery, family history of breast cancer, recurrence time, recurrence, metastasis time, metastasis, tumor grade, and treatment. The second dataset contained 72 patients with similar variables. This study was approved by the Institutional Review Board of Isfahan University of Medical Sciences (Ethical code: IR.MUI.REC.1401.355). The study was conducted in accordance with the Declaration of Helsinki. The two datasets were merged to form a single dataset containing 142 patients; four patients were excluded due to incomplete information. Variables with more than 25% missing data, such as menopausal status and cancer stage, were excluded. Baseline clinical and pathological variables were considered, including age, tumor size, nodal status, histological grade, Ki-67, type of surgery, chemotherapy, and radiotherapy. Only variables available at the time of diagnosis were included to prevent immortal time bias, and all follow-up events (e.g., recurrence, recurrence time) were excluded from the predictors.

For binary variables, the absence of a variable was coded as 0, and the presence of a variable was coded as 1. Age was dichotomized, with patients under 58 years coded as 0 and those 58 years or older as 1. For Ki-67, values below 35% were coded as 0, while values of 35% or higher were coded as 1. Tumor size was categorized into two groups, smaller and larger than 5 cm, coded as 0 and 1, respectively. The type of surgery was recorded as 0 for lumpectomy and 1 for radical mastectomy. Grades G1 and G2 were grouped and coded as 0, while grade G3 was coded as 1. Finally, treatment was categorized into chemotherapy alone, coded as 0, and chemotherapy combined with radiotherapy, coded as 1. Two of the total three variables were assessed using the logical AND operator; this means that if an individual has both variables present, it is coded as 1, and if one of the variables is absent, it is coded as 0. For example, the variable LymphXtreatment is coded as 1 if both lymph and treatment are present; the same logic applies to MetastasisXgrade. Patients were followed from diagnosis to death or last follow-up; individuals without an observed event were treated as right censored.

**Risk function.** To estimate the risk function and assess the relationship between the clinical and demographic factors and survival outcomes, we used CoxPH regression models in the *R* programming language. The CoxPH model enables the estimation of HRs while accounting for censored data. The CoxPH model is described using Equation 1 [19, 20]:

(1) $$h\left(t|X\right)\;=\;h_{0}\left(t\right)\;\times {\;e}^{\left(\beta_{1}x_{1}\;+\;\beta_{2}x_{2}+\ldots +\;\beta_{n}x_{n}\right)}$$

where:

$h\left(t|X\right)$ is the hazard function at time *t*;

$h_{0}\left(t\right)\;$ is the baseline hazard function at time *t* (i.e., the hazard when all the covariates are zero);

$X\;=\;[x_{1},x_{2},\ldots ,x_{n}]$ are the predictors variables;

$\beta_{1},{\;\beta }_{2},\ldots ,\;\;\beta_{n}$ are the coefficients of the predictor variables.

Regression models were applied to a set of risk factors. To minimize the risk of overfitting given the limited number of outcome events (34 deaths), we restricted the Cox model to six clinically established predictors: age, treatment type, grade, lymph node status, metastasis at diagnosis, and type of surgery. To obtain the relative risk for a given individual, we compute the “risk” type formula of the CoxPH function. This value is a proportion of an event occurring in relation to the baseline. The risk is calculated as shown in Equation 2:

(2) $$Absolute\;Risk\;\left(t|X\right)\;=\;1\;-\;exp(-\int_{0}^{t}h\left(s|X\right)ds)$$

A value over one indicates a higher likelihood of the event occurring for an individual, while a value under one suggests a reduced chance of the event happening [17, 18].

### Internal validation

To evaluate the validity and predictive accuracy of our model, we implemented the stratified five-fold cross-validation. In this approach, the dataset is randomly divided into five groups while preserving the proportion of events within each group. In each iteration, 80% of the data for training the model, and the remaining 20% is reserved for testing. This procedure was repeated for each of the five groups in the dataset so each fold served once as a test set. Model discrimination was quantified using the Harrell’s C-index [21], the standard metric for Cox models. Model discrimination at a prespecified 5-year horizon (60 months) was evaluated using the inverse probability of censoring weighted (IPCW) [22] time-dependent AUC. At time *t*, the time-dependent AUC is defined in Equation 3 [23–26]:

(3) $$AUC\left(t\right)\;=\;P\left({\hat{r}}_{i}\;>\;{\hat{r}}_{j}\right|T_{i}\;\leq \;t,T_{j}\;>\;t)$$

where ${\hat{r}}_{i}\;=1\;-\;\hat{S}\left(t|x_{i}\right)$ denotes the predicted risk at time *t*, and IPCW was used to account for right censoring. Prediction error was assessed using the IPCW-adjusted Brier score at 60 months as defined in Equation 4 [27].

(4) $$Brier\left(t\right)\;=\;E\left[w_{i\left(t\right)}{\left(I\left(T_{i}\;\leq \;t\right)\;-\;{\hat{r}}_{i}\right)}^{2}\right]$$

where I(⋅) is the event indicator and $w_{i\left(t\right)}$ denotes inverse probability of censoring weights estimated from the Kaplan-Meier censoring distribution. All time-dependent performance metrics were computed in *R* using the timeROC and riskRegression packages and MedCalc software Ltd [28]. We assessed calibration graphically using a calibration plot with ‘calibrate’ function in ‘rms’ package in *R* [29–32]. Calibration curves were internally validated using bootstrap resampling (B = 200).

To evaluate calibration at the prespecified 5-year horizon (60 months), we calculated the overall bias, defined as the relative difference between the predicted and observed incidence in Equation 5 [33, 34]:

(5) $$Overall\;Bias\;\left(\%\right)\;=\;\left(\frac{Predicted\;event\;-\;Observed\;event}{Observed\;event}\right)\;\times \;100$$

To evaluate whether the model performs equally across different subgroups, accuracy, true positive rate (TPR) and false positive rate (FPR) were calculated. Accuracy measures the overall ratio of correctly classified individuals among all predictions. Equation 6 indicates the performance of the model:

(6) $$Accuracy\;=\;\frac{TP\;+\;TN}{FP\;+\;FN\;+\;TP\;+\;TN}$$

where TP is the number of correctly predicted positives, TN is the number of correctly predicted negatives, FP is the number of incorrectly predicted positives, and FN represents incorrectly predicted negatives by the model [35].

TPR, indicating the ratio of actual positive cases that are correctly identified by the model, is estimated as defined in Equation 7 [36]:

(7) $$TPR\;=\;\frac{TP}{TP\;+\;FN}$$

Finally, FPR quantifies the ratio of negative cases that are incorrectly classified as positive, Equation 8 reflecting the model’s tendency to produce false alarms [36]:

(8) $$FPR\;=\;\frac{FP}{FP\;+\;TN}$$

To estimate uncertainty, 95% confidence intervals (CIs) for the given metrics were computed using *the R* programming language (*R* version 4.4.2). For Harrell’s C-index, CIs were obtained from the cross-validation distribution of the performance estimates. For receiver operating characteristic area under the curve (AUROC), 95% CIs were calculated using the nonparametric DeLong method as implemented in *R* packages.

**Figure 1:**
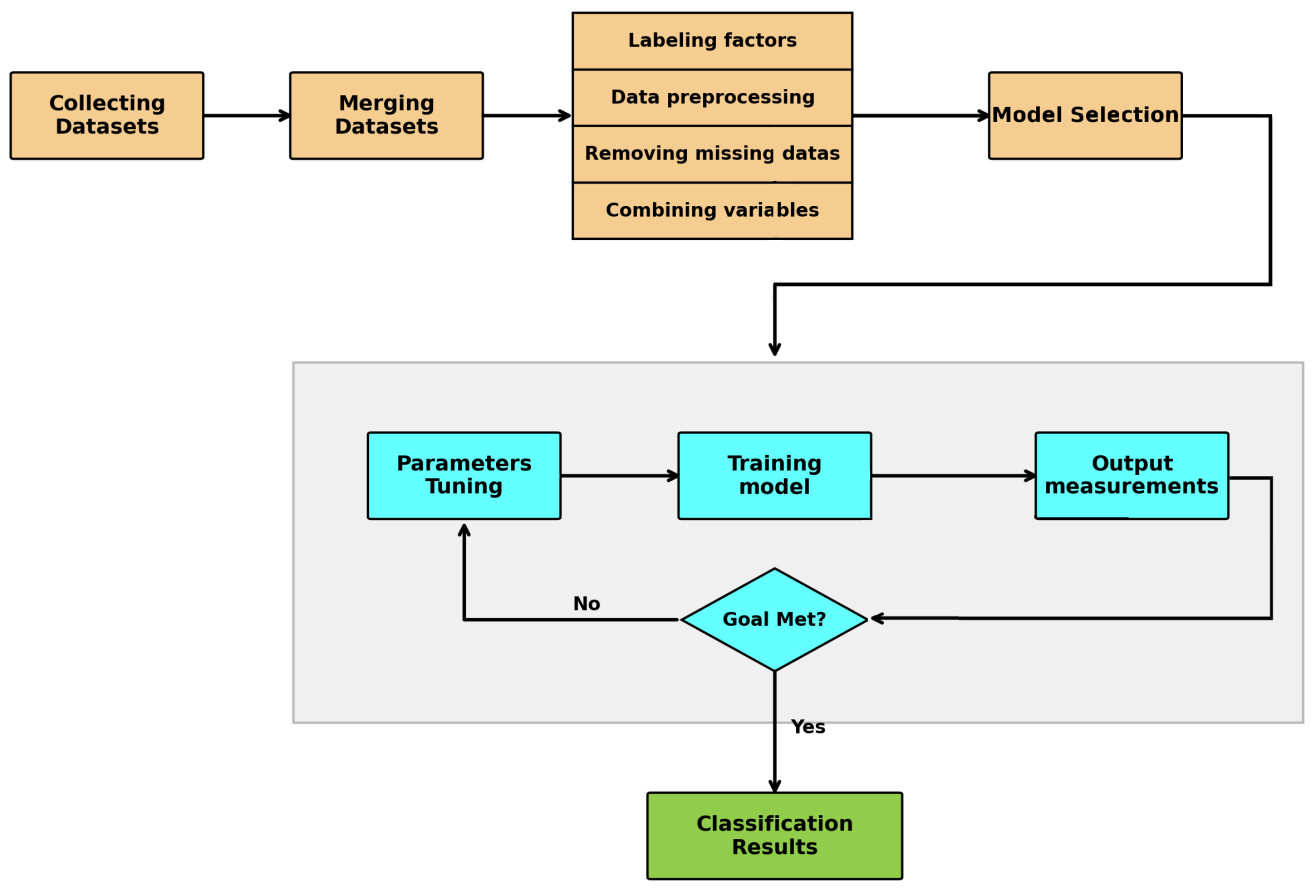
An overview of the steps to achieve the proposed method.

Figure 1 shows the overall framework of the proposed method. The process begins with gathering data until the classification results are obtained.

We used TPR and FPR to assess fairness of the prediction model. Equality of opportunity is assessed by comparing the TPR values between groups. Individuals who truly belong to the positive class must have an equal chance of being correctly identified across groups. For the model ϒ and outcome of Y, it is estimated as defined in Equation 9:

(9) P(ϒ = 1|Y = 1,  A = m) = P(ϒ = 1|Y = 1,  A = n)

where A is the sensitive attribute with *m* and *n* values [37].

Also, predictive equality focuses on ensuring that FPR is equal across groups. No group must unfairly be subjected to a higher rate of incorrect positive predictions. For the same model, predictive equality is defined as Equation 10 [37]:

(10) P(ϒ = 1|Y = 0,  A = m) = P(ϒ = 1|Y = 0,  A = n)

Demographic parity is the next fairness criterion in evaluating the fairness of an artificial intelligence model. It ensures that all groups have equal rates of positive predictions. This criterion is defined as Equation 11 [37]:

(11) P(ϒ = ϒ'∣A = m)=P(ϒ = ϒ'∣A = n)

We check whether the model satisfies the equality of odds criterion. It tests if the model’s sensitivity and specificity are consistent across subgroups. It is estimated as defined in Equation 12:

(12) $$Pr\left\{ϒ\;=\;ϒ'|Y\;=\;y,\;\;A\;=\;m\right\}\;=\;Pr\;\left\{ϒ\;=\;ϒ'|Y\;=\;y,\;\;A\;=\;n\right\}$$

where A is the sensitive attribute for all values [38]. Equalized odds ensure that TPR and FPR are similar across groups. The positive class was defined as death within 5 years of diagnosis (Y = 1), and the negative class as survival beyond 5 years (Y = 0).

### Statistical methods

Bias in the model was assessed following the guidelines of ISO/IEC TR 24027:2021 [37], which focuses on detecting and reducing bias in artificial intelligence decision systems. To detect statistically significant differences between groups and also variables, *Z* tests were applied to achieve the *P* values. Effect sizes were calculated to quantify the magnitude of bias using Cohen’s *h* for TPR and FPR differences across subgroups and variables respectively. This approach evaluates the agreement between observed and predicted probabilities. All statistical tests were performed using IBM SPSS Statistics version 27 [39].

Because the Hosmer-Lemeshow test is not applicable to CoxPH models, model calibration was instead assessed at pre-specified horizons (5 years) by comparing predicted risks with observed Kaplan-Meier estimates across risk quantiles. For each variable included in the final model, the correlation between data and follow-up time was tested. The proportional hazards (PH) assumption of the CoxPH model was assessed with Schoenfeld residuals [40]. In a CoxPH model, the HR for a predictor must be constant over time. The package ‘survival’ [32] in the *R* programming language uses the correlation over time and with statistical tests, hypotheses, and violation of PH was calculated.

## Results

All variables are summarized in Table 2. The *P*-values and effect sizes stated below were achieved from the Z tests and Cohen’s *h*, respectively. A total of 138 patients were enrolled in the study, among whom 34 (24.6%) died, and 104 (75.4%) survived. The mean age of the cohort was 54 years. Of the Ki-67 expression, 62.6% of patients exhibited levels above 35%, 31.9% had levels between 15% and 35%, and only 5% had Ki-67 values below 15%. A history of comorbid conditions such as diabetes, cardiovascular disease, and hypertension was reported in 38.4% of patients, most of whom were among the surviving group. Lymph node involvement was observed in 33.4% of patients, with a relatively even distribution between deceased and surviving individuals. In contrast, tumor size differed significantly between groups (*P* < 0.01). 61.6% of patients had tumors measuring between 2 cm and 5 cm, while 11.6% had tumors larger than 5 cm, the majority of whom were in the deceased group. The type of surgery was also significantly associated with survival outcomes (*P* < 0.01, effect size = 0.28). While radical mastectomy was more common among those who died (55.9%), lumpectomy was more common among survivors (74%). Family history of breast or other cancers was reported in 55.8% of the cohort. Disease recurrence occurred in 5.8% of patients and was significantly associated with mortality (*P* = 0.03). Metastatic disease was strongly associated with death; 76.5% of patients who died had metastases, whereas only 6.7% of survivors did (*P* < 0.01). Overall, metastasis was present in 23.9% of the study population. The majority of patients had high tumor grades; 73.3% of patients were diagnosed with grade 3 tumors. Regarding treatment, 86.2% of patients received both chemotherapy and radiotherapy, and this combined approach was significantly associated with better survival outcomes (*P* < 0.01).

**Table 2:** The variables with their distributions, effect sizes, and *P*-values.

**Variables**	**Overall (*N* = 138)**	**No (*N* = 104)**	**Yes (*N* = 34)**	***P*-value**	**Effect size**
**Age**					
Mean (SD)	54.0 (12.5)	54.2 (11.9)	53.4 (14.4)	0.76	0.06
Median [Min, Max]	53.0 [33.0, 85.0]	53.0 [33.0, 81.0]	51.5 [33.0, 85.0]		
**Ki-67**					
< 15%	7 (5.1%)	4 (3.9%)	3 (8.8%)	0.30	0.13
> 35%	87 (63.0%)	69 (66.3%)	18 (52.9%)		
15–35%	44 (31.9%)	31 (29.8%)	13 (38.2%)		
**History of comorbid conditions**					
No	85 (61.6%)	61 (58.6%)	24 (70.6%)	0.27	0.11
Yes	53 (38.4%)	43 (41.4%)	10 (29.4%)		
**Lymph**					
No	92 (66.6%)	70 (67.3%)	22 (64.7%)	0.99	0.02
Yes	46 (33.4%)	34 (32.7%)	12 (35.3%)		
**Tumor size**					
< 2 cm	37 (26.8%)	29 (27.9%)	8 (23.5%)	< 0.01	0.27
> 5 cm	16 (11.6%)	7 (6.7%)	9 (26.5%)		
2–5 cm	85 (61.6%)	68 (65.4%)	17 (50.0%)		
**Type of surgery**					
Lumpectomy	91 (65.9%)	77 (74.0%)	15 (44.1%)	< 0.01	0.28
Radical mastectomy	47 (34.1%)	27 (26.0%)	19 (55.9%)		
**BC family**					
No	61 (44.2%)	45 (43.3%)	16 (47.1%)	0.89	0.03
Yes	77 (55.8%)	59 (56.7%)	18 (52.9%)		
**Recurrence**					
No	130 (94.2%)	101 (97.1%)	29 (85.3%)	0.03	0.22
Yes	8 (5.8%)	3 (2.9%)	5 (14.7%)		
**Metastasis**					
No	105 (76.1%)	97 (93.3%)	8 (23.5%)	< 0.01	0.70
Yes	33 (23.9%)	7 (6.7%)	26 (76.5%)		
**Grade**					
G1	9 (6.5%)	7 (6.7%)	2 (5.9%)	0.90	0.04
G2	28 (20.2%)	20 (19.2%)	8 (23.5%)		
G3	101 (73.3%)	77 (74.0%)	24 (70.6%)		
**Treatment**					
Chemotherapy	19 (13.8%)	8 (7.7%)	11 (32.4%)	< 0.01	0.31
Chemotherapy + radiotherapy	119 (86.2%)	96 (92.3%)	23 (67.6%)		

**Figure 2:**
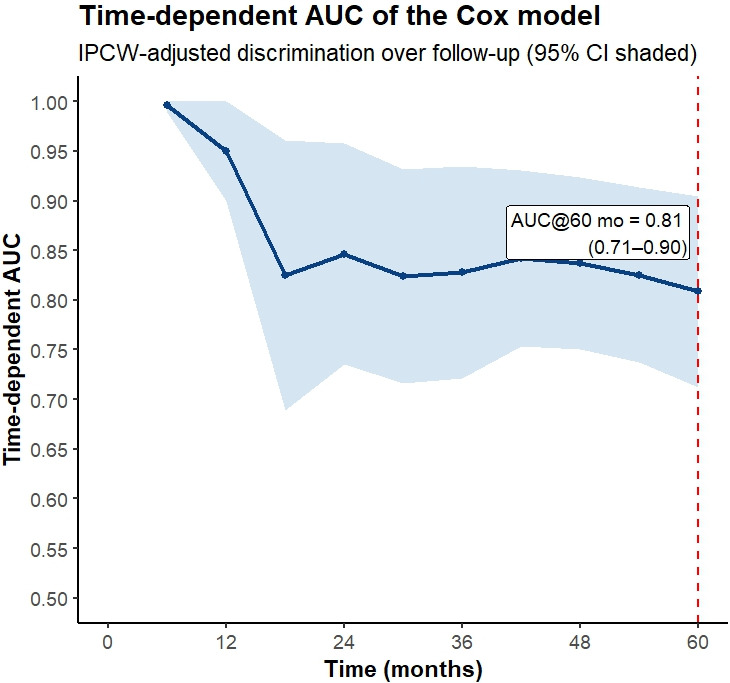
**Time-dependent AUC curve for the Cox model with 95% confidence intervals (IPCW-adjusted).** The dashed line indicates the 5-year horizon (60 months). AUC: area under the curve; IPCW: inverse probability of censoring weighted.

We used CoxPH regression analysis in *R* version 4.4.2 [41] to evaluate several predictors and their interactions; several baseline factors were found to be significantly associated with TNBC-related events. These factors included age (binary with threshold of 58 years old), type of surgery, grade × metastasis (the relation between tumor grade and metastasis by logic AND coded), and lymph node × treatment (the relationship between lymph node involvement and treatment type by logic AND coded). Variables such as Ki-67 and tumor size were available in the dataset but were not included in the final Cox model. The final model demonstrated strong discriminatory performance in a five-fold stratified cross-validation, with a Harrell’s C-index of 0.80 (95% CI: 0.72–0.87) and a time-dependent AUC of 0.81 (95% CI: 0.72–0.90) at 5 years. Figure 2 displays the time-dependent AUC, which remained high throughout follow-up. At the prespecified 5-year horizon (60 months), the IPCW time-dependent AUC was approximately 0.81, indicating good discrimination for 5-year mortality risk. Moreover, the model predicted 31 events, whereas 34 events were observed in the cohort. This corresponds to an overall bias of –8.8%, indicating that the model slightly underestimated the occurrence of events. The estimated coefficients and corresponding HRs for the variables included in the CoxPH model are presented in Table 3.

**Table 3:** The Cox model results, including regression coefficients and HRs with their 95% CI.

**Variable**	**Coefficient (β)**	**Hazard ratio (HR)**	**95% CI for HR**	***P*-value**
Age	0.719	2.054	0.955–4.418	0.06
Type of surgery	1.059	2.884	1.277–6.513	0.01
Lymph × treatment	–1.292	0.275	0.094–0.798	0.02
Metastasis × grade	1.960	7.101	3.349–15.057	< 0.01

CI: confidence interval.

At the 5-year horizon (60 months), the IPCW with adjusted Brier score was 0.131 (95% CI: 0.084–0.178), improving upon the null model (0.171). The index of prediction accuracy (IPA) was 23.5%, indicating a 23.5% reduction in prediction error compared to the null model.

**Figure 3:**
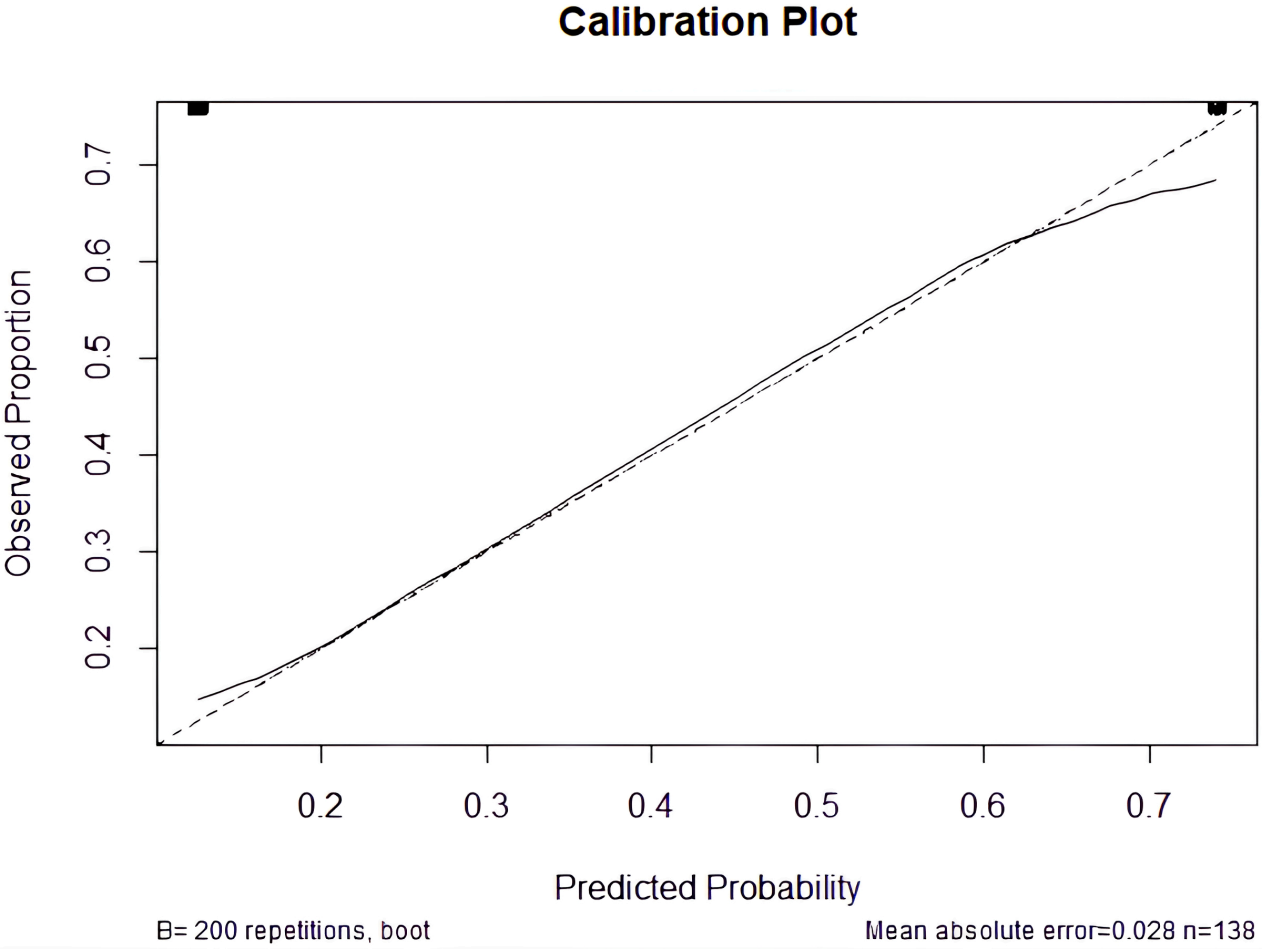
**Calibration plot comparing predicted probabilities with observed outcomes.** The dashed diagonal line represents perfect calibration (*R*^2^ = 0.36).

To assess the calibration of the predictive model, a calibration curve was generated to evaluate the agreement between predicted probabilities and observed outcomes. This approach enabled the visual examination of how closely the model’s predictions aligned with the rates of actual events [30]. At 5 years, the calibration curve showed good agreement between predicted and observed survival probabilities. The resulting curve is presented in Figure 3.

In the demographic parity analysis, the variable age did not show a statistically significant difference between groups (*P* = 0.29). The observed effect size was small (Cohen’s *h* = 0.18), which shows minimal practical difference across age categories. Likewise, lymph variable showed no statistically significant difference between groups (*P* = 0.24) with a small effect size (Cohen’s *h* = 0.20). Therefore, the model’s predictions were largely independent of lymph status.

In the equality of opportunity analysis, three variables showed statistically significant differences across groups. The variable treatment had a *P*-value of 0.15 with a medium effect size (Cohen’s *h* = 0.55). The variable age (*P* = 0.30, Cohen’s *h* = 0.37) and lymph (*P* = 0.35, Cohen’s *h* = 0.34) also demonstrated no significant differences with small effect sizes, indicating that the TPRs were relatively consistent across these subgroups.

We used the L2 distance to measure the difference between TPR and FPR across subgroups. The variables age and lymph showed a relatively small disparity with L2 distances of 0.22 and 0.16, respectively. Variable type of surgery exhibited a higher level of disparity (L2 distance = 0.46), indicating that this variable influences the model’s prediction more noticeably.

For the last criterion of fairness analysis, two variables showed statistically significant differences in FPRs. Variable age was significantly associated (*P* = 0.02) with a medium effect size (Cohen’s *h* = 0.45), which means a potential disparity in prediction error across age categories. Similarly, the type of surgery had a significant result (*P* = 0.03) with a medium effect size (Cohen’s *h* = 0.43). Therefore, the model may systematically produce higher FPRs for certain surgical groups.

The PH assumption using Schoenfeld residuals for all variables included in the proposed model, as shown in Table 4, the HRs were constant over time. *P*-values above 0.05 suggest that no violations were observed. However, the surgery type showed borderline evidence of non-proportionality (*P* = 0.05).

**Table 4:** The statistical results of the proportional hazard assumption of the model.

**Variable**	***P*-value**
Age	0.42
Type of surgery	0.05
Lymph × treatment	0.58
Metastasis × grade	0.21

**Figure 4:**
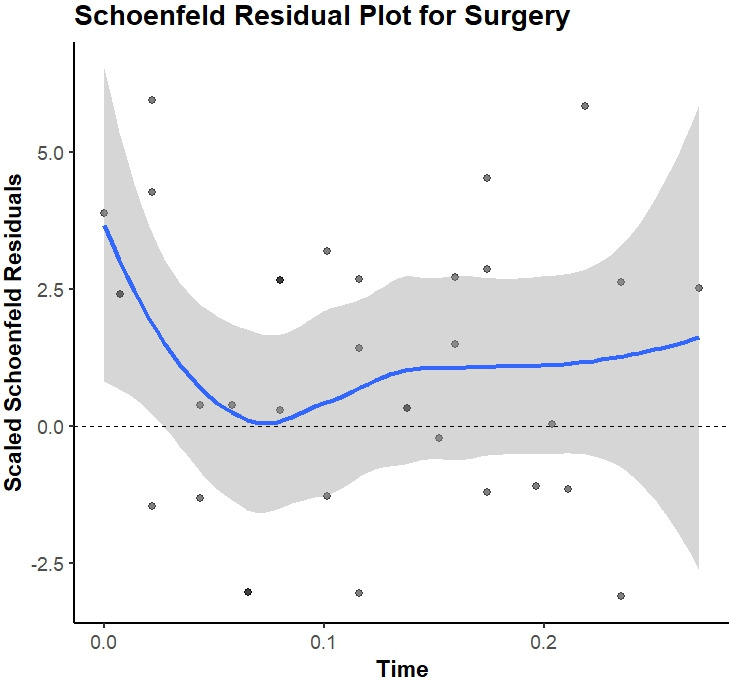
Schoenfeld residual plot of variable “Type of surgery”.

Schoenfeld residual plot of the variable “Type of surgery” is provided in Figure 4. The PH assumption held for all covariates; surgery type showed borderline deviation (*P* ≈ 0.05). In a stratified Cox sensitivity analysis (strata = surgery type), coefficients for other predictors remained similar.

## Discussion

### Interpretation

The developed CoxPH model confirmed metastasis at diagnosis, high tumor grade (G3), and type of surgery (radical mastectomy vs. lumpectomy) as the dominant prognostic factors for TNBC in this MENA cohort. Patients who presented with distant metastasis at baseline had a substantially higher hazard of death, which is expected since metastatic disease (stage IV) portends poor survival. Similarly, those with grade 3 tumors faced worse outcomes than those with grade 1–2, consistent with the aggressive biology of high-grade TNBC tumors. The finding that patients undergoing radical mastectomy had higher mortality than those treated with breast-conserving surgery likely reflects indication bias—i.e., patients requiring mastectomy had more advanced local disease—rather than the surgical procedure itself being detrimental. Other baseline variables included in the model (age, lymph node involvement, and treatment modality) were not significantly associated with survival in this cohort. Notably, age (dichotomized at 58 years) did not emerge as an independent predictor, in contrast to larger studies where younger age has correlated with better TNBC outcomes. This discrepancy may be due to the relatively small sample and binary age grouping, which could mask gradations of age effect observed in broader populations. Overall, the model’s performance was strong, with a Harrell’s C-index of 0.80 and AUROC of 0.81, indicating excellent discrimination between survivors and non-survivors. The calibration analysis showed close agreement between predicted and observed 5-year survival probabilities (within ~9% difference), suggesting the risk estimates are well-calibrated. In practical terms, a patient predicted to have (for example) an 80% 5-year survival by the model would on average have an actual survival probability in the 70–80% range in our data. This level of accuracy implies the model reliably stratifies patients by risk. Finally, the fairness assessment revealed mostly equitable performance across subgroups. The model’s error rates were similar for patients with and without lymph node involvement and across other clinical subgroups, indicating no major bias in those dimensions. Minor disparities in false-positive rates were observed between age groups and surgical types—for instance, one subgroup (such as older patients or those receiving a particular surgery) had a slightly higher false-alarm rate—but these differences were small in magnitude. Importantly, there was no evidence of systemic bias favoring or harming a particular patient group; the sensitivities (TPRs) remained comparable, satisfying the fairness criteria to a reasonable extent. In summary, the interpretation of our results is that the Cox model successfully identified intuitive prognostic factors and achieved robust predictive performance without introducing significant unfairness between key patient subpopulations.

### Proportional hazards and surgery type

The Schoenfeld residual test for the type of surgery yielded a borderline *P*-value of approximately 0.05, and the residual plot indicated a mild deviation from proportionality. This suggests that the effect of surgery may vary over time. This is clinically plausible because surgery type often serves as a proxy for baseline disease burden and treatment indication (e.g., larger tumors or more extensive disease leading to mastectomy). Its association with mortality tends to be stronger early after diagnosis and weaker later on. To assess robustness, we performed a sensitivity analysis using a stratified Cox model, defining the strata by surgery type. This approach relaxes the PH assumption for the surgery type covariate by allowing for group-specific baseline hazards. The estimated effects of the remaining predictors and the overall conclusions remained largely unchanged, which supports the stability of our main findings despite the potential for non-proportionality in surgery.

### Mitigation strategies

We observed moderate disparities in FPRs across age and surgery subgroups. A higher FPR in older patients and in one surgery subgroup indicates that these patients may be more frequently classified as high-risk despite not experiencing 5-year mortality. Several mitigation approaches could be considered to reduce subgroup disparities, particularly in FPR. First, threshold adjustment (group-specific decision thresholds) could be used to equalize FPR across age and surgery groups at the 5-year horizon. Second, calibration within subgroups (recalibration of baseline survival or risk mapping) may reduce systematic overestimation of risk in groups with higher FPR. Third, cost-sensitive learning or reweighting during model fitting could penalize false positives more strongly in groups with higher observed FPR. Finally, because surgery type may partially reflect baseline disease severity and treatment indication, future multicenter external validation is needed to determine whether this disparity represents a true transportability issue or center-specific clinical practice patterns.

### Comparison with the state of the art

Our clinical research focuses on predicting survival risk over a 5-year time horizon using commonly available clinical and pathological features, which is in line with the advancement of imaging and diagnostic AI systems and the development of new methods such as hyperspectral imaging for breast cancer diagnosis [42, 43]. Our findings align with and extend the current state of TNBC prognostic modeling. Prognostic factors: The key risk factors highlighted by our model—extent of disease (metastasis), tumor grade, and to a lesser degree nodal status and age—are broadly consistent with those identified in prior studies. For example, Zarean Shahraki et al. [5] reported that tumor stage and lymph node involvement were among the strongest predictors of TNBC outcomes, and Zhou and Chen [6] found that lower stage (absence of metastasis) and lower grade were associated with significantly better survival. These parallels reinforce that our model is capturing fundamental prognostic signals of TNBC. One notable difference is that age was not significant in our cohort, whereas larger population-based studies (e.g., SEER analyses) have observed that younger patients tend to have modestly better TNBC survival. It is possible that our dichotomization (under vs. over 58 years) and limited sample size reduced sensitivity to age effects, or that in a MENA context, age plays a less pronounced role due to different comorbidities or treatment patterns. Likewise, we included the type of surgery as a predictor and found a survival disparity between mastectomy and lumpectomy recipients, which, to our knowledge, has not been a focus of other TNBC nomograms. Large database studies usually emphasize whether surgery was performed at all (showing surgery confers a survival benefit), but not the extent of surgery. Our result likely reflects case-mix (more extensive surgery for more advanced tumors) rather than a causal effect; nonetheless, it underscores the importance of baseline tumor burden in outcomes.

Model performance: In terms of predictive accuracy, our model is competitive with, and in some cases exceeds, previously published models despite the smaller scale of our study. The C-index of 0.80 we achieved is on par with the best-performing TNBC prognostic models in the literature. For instance, a recent Cox-based model by Zhou and Chen [6] attained an internal C-index of ~0.79, and Guo et al. [15] reported ~0.78–0.80 in internal validation of their nomogram. Many other nomograms and machine learning models for TNBC have shown C-indices in the 0.69–0.75 range, suggesting our model’s discrimination is at the upper end of reported performance. The AUROC of 0.81 in our study similarly falls in a high range; by comparison, Meng et al. [7] (using a LASSO-based prognostic model) reported AUC values from 0.75 up to 0.86 across different time horizons, and a machine learning ensemble by Huang et al. [10] achieved an AUC of ~0.81. This comparison indicates that our relatively simple six-variable Cox model —carefully validated via cross-validation—can perform on par with more complex approaches that leverage dozens of features or advanced algorithms. The advantage of our approach is that it retains clinical interpretability (via HRs for known risk factors) while still delivering accuracy comparable to black-box models like neural networks [5] or gradient boosting [10]. Such interpretability is crucial in clinical settings for gaining physician trust and understanding model decisions.

Fairness and novel contributions: A distinctive aspect of our work is the integration of an algorithmic fairness assessment, which to our knowledge, has not been addressed in prior TNBC survival studies. None of the published models [5–16] explicitly evaluated whether their predictions were equitable across patient subgroups—an oversight our study begins to fill. We applied fairness metrics grounded in the ISO/IEC TR 24027:2021 standard for AI bias [32] as well as concepts like equality of opportunity in supervised learning [33]. By doing so, we demonstrated that high performance can be achieved without introducing large biases between groups (e.g., older vs. younger patients). This goes beyond the state-of-the-art of TNBC prognostic models, which have traditionally focused only on accuracy (discrimination and calibration) but not on the ethical dimension of model performance. In the broader context, fairness in clinical predictive modeling is an emerging concern, and our study is among the first in oncology to prospectively audit a model for bias using formal criteria. This comparison underscores that our work not only matches contemporary models in predictive power but also introduces a new standard for model evaluation. In summary, relative to the state-of-the-art, our model stands out as clinically parsimonious, predictively robust, and fairness-aware, addressing gaps that previous studies had left open (such as the under-representation of MENA populations and the lack of bias assessment).

### Limitations

Despite its strengths, this study has several limitations that must be acknowledged. First, the sample size (*N* = 138) is relatively small and drawn from a single regional population. In contrast, many TNBC prognostic models have been derived from large databases with thousands of patients or multi-center cohorts, and often include an external validation set. Our internal cross-validation provides some confidence in the model’s generalizability, but the lack of an independent external cohort means the model may be overfit to the idiosyncrasies of our dataset. The performance metrics we report (C-index 0.80, AUC 0.81) were achieved on internal resampling; an external test could yield lower values, as seen in other studies where C-indices dropped a few points on validation cohorts. Thus, caution is needed in extrapolating our results to other settings, and external validation in a separate MENA TNBC cohort (or a subset of a larger registry) is a critical next step.

Second, there are limitations in the variables and data availability. We restricted our model to baseline clinical and pathological features that were available for all patients, which meant excluding some potentially important factors due to missing data or study design. For example, cancer stage (beyond metastatic vs. non-metastatic) was not directly included because detailed TNM staging data were incomplete for many patients. By encoding only the presence of metastasis, our model captures the extreme of stage IV disease but cannot distinguish, say, stage II vs. stage III—finer prognostic distinctions that could be relevant. Similarly, menopausal status and other comorbid conditions were omitted due to data sparsity. While this ensured a clean dataset, it may omit prognostic information. Additionally, our treatment variable was simplified to whether radiotherapy was added to chemotherapy. We did not differentiate patients by surgical approach beyond lumpectomy vs. mastectomy, nor by systemic therapy details (e.g., neoadjuvant vs. adjuvant chemotherapy, use of newer agents). In practice, treatment timing and modalities can influence outcomes [11]; for instance, Haiderali et al. [11] observed different survival rates depending on whether patients received neoadjuvant therapy, surgery, both, or neither. Our analysis does not account for these nuances, representing a potential confounding factor if, for example, more aggressive treatments were given to higher-risk patients.

Third, we did not incorporate any tumor biological markers. TNBC is known to be a heterogeneous disease with emerging molecular subtypes and genomic differences that could impact prognosis [2]. Prior research has identified distinct molecular stratifications within TNBC that correlate with outcomes [2], and novel prognostic biomarkers (e.g., gene expression profiles or immune markers) are an active area of investigation. Our model, by design, used only routinely available clinicopathologic factors; this makes it broadly applicable but means it might not capture all facets of tumor behavior. The absence of more granular tumor biology or laboratory biomarkers (such as BRCA mutation status, basal vs. non-basal subtype, or inflammatory markers) is a limitation. Similarly, socio-demographic factors like race, socioeconomic status, or marital status were not included. There is evidence that marital status can influence cancer outcomes (possibly via support networks and treatment adherence), and other social determinants might affect survival, especially in diverse populations. Our cohort was relatively homogeneous in this regard (all patients treated in similar institutions within one region), so we had limited ability to examine such factors.

From a methodological standpoint, the Cox model assumptions and design choices impose some limitations. We assumed PH for all included covariates; tests for the PH assumption did not show significant violations, though one covariate (surgery type) was borderline (*P* ≈ 0.05 for non-proportionality). If the HR for surgery changes over time (e.g., perhaps the early survival advantage of lumpectomy vs. mastectomy diminishes at later follow-up), our model might not capture that time-varying effect. More flexible modeling (such as including time-dependent covariates or using stratified Cox models) could address this, but we opted for simplicity given the sample size. We also dichotomized continuous predictor variable age and grade for clinical interpretability, using established cut-points. While this simplification eases use of the model, it can reduce statistical power and granularity —for instance, two patients aged 58 and 60 are classed into different risk groups in our model, even though their age difference is small. A model treating age as a continuous or multi-category variable might detect a trend that our binary split could not. Lastly, although we performed a comprehensive fairness evaluation, we acknowledge that our analysis was limited to the attributes collected (age, surgery type, nodal status, etc.) and the definitions provided by the ISO standard. We did not assess intersectional fairness (e.g., combinations of factors) or attempt to mitigate the slight disparities observed. The threshold for “notable” bias in false-positive rates was not rigorously quantified; we qualitatively noted differences, but a larger sample would be needed to statistically confirm if those deviations are significant. Moreover, fairness metrics can sometimes trade off with model accuracy—a model optimized to equalize false-positive rates between groups might sacrifice some overall discrimination [33]. We did not explore such trade-offs here, focusing instead on measuring bias with the model as-is. This is a limitation in the sense that while we flagged minor biases, we did not adjust the model to eliminate them. Future work could consider cost-sensitive adjustments if those disparities are deemed clinically important.

Despite key strengths—namely the use of a clinically interpretable Cox model built on routinely available baseline variables, censoring-aware evaluation at a prespecified 5-year horizon (IPCW time-dependent AUC/Brier score), explicit calibration assessment, and a formal fairness audit aligned with ISO/IEC TR 24027:2021—this study’s fairness conclusions remain constrained by the single-center design and the available sensitive-attribute axes: the cohort (*N* = 138; 34 events) comes from one MENA institution, so it cannot directly test MENA-relevant disparities driven by cross-country differences, urban-rural residence, insurance status, or public vs. private care pathways, and the modest sample size limits power for fine-grained subgroup and intersectional analyses; consequently, while we can quantify parity, calibration, and error-rate differences across recorded subgroups (e.g., age and surgery type) and transparently report moderate FPR disparities, broader MENA inequity dimensions require multicenter data with explicit socioeconomic/geographic variables (or validated proxies) and external validation to confirm transportability and to distinguish true bias from center-specific practice patterns.

### Implications and future work

Clinical implications: The prognostic model presented in this study has practical implications for TNBC management, particularly in Middle East-North Africa settings. By relying on routine diagnostic variables readily available at diagnosis (age, basic tumor metrics, etc.), the model can be integrated into clinical workflows without the need for special tests. Oncologists could use the predicted risk (e.g., 5-year mortality risk) to stratify patients into different surveillance or treatment intensity groups. For example, a TNBC patient identified as high-risk (perhaps due to a grade 3 tumor with baseline metastasis) could be considered for more aggressive or novel therapies and closer follow-up, whereas a low-risk patient might avoid overtreatment. Such risk-adaptive strategies align with personalized medicine goals and could ultimately improve outcomes. Importantly, our incorporation of fairness metrics enhances clinical trust in the model: stakeholders can be reassured that the tool was vetted for equitable performance across subgroups. In practice, this means a clinician can feel more confident that the model’s recommendations do not inadvertently favor one type of patient over another (for instance, that it isn’t systematically under-predicting risk in older patients or over-predicting for those who had lumpectomies). As algorithmic decision-support tools begin to enter healthcare, this kind of fairness audit will be increasingly expected by regulatory bodies and end-users. Our study serves as a proof-of-concept that such audits are feasible in oncology. The transparent reporting of model bias (or lack thereof) can help in building patient trust as well—patients are more likely to accept AI-driven recommendations if they know the model has been checked for biases that could affect them.

Policy and regional implications: This work also contributes local evidence to guide breast cancer care in the MENA region. TNBC outcomes and patient characteristics in MENA can differ from Western populations due to genetic, environmental, and healthcare-system factors, yet previously most prognostic models were developed on Western cohorts. Our results help fill this gap by providing data on what factors drive survival specifically in a MENA cohort and demonstrating that a model built on regional data can achieve high accuracy. This could encourage the development of region-specific or institutional prognostic tools elsewhere, especially in regions underrepresented in global cancer research. Additionally, having a validated risk prediction model supports health system planning—for instance, identifying that a quarter of TNBC patients here died within ~5 years (24.6% mortality) emphasizes the need for improved therapies and perhaps earlier detection in our setting. Our approach also dovetails with global initiatives like the WHO’s GBCI, which aims to reduce breast cancer mortality through strengthening health systems and tailored interventions. By quantifying risk and highlighting subgroups (like metastatic presentations) with especially poor survival, our model can help target resources in line with such initiatives (e.g., improving metastatic TNBC care pathways to meet mortality reduction goals).

Future research directions: Building on this work, several avenues for future research are recommended. External validation is a top priority: applying our model to an independent TNBC patient cohort (ideally from another center or country in the region) to test its calibration and discrimination. This will confirm if the model generalizes or if recalibration is needed for different settings. We plan to seek collaborations with other MENA oncology centers to validate and possibly update the model with larger data. Additionally, prospective studies could evaluate the model’s utility in real-time clinical decision-making—for example, a trial where the model’s risk estimates are provided to tumor boards to see if it improves treatment personalization or patient outcomes.

Another future direction is to incorporate additional predictors to further enhance prognostic accuracy. As data availability improves, one could integrate genomic or molecular features of TNBC (e.g., BRCA mutation status, gene expression profiles) into the model, potentially using a hybrid approach that retains interpretability for clinical variables but adds a genomic risk score. Similarly, incorporating treatment response indicators such as pCR after neoadjuvant therapy (where applicable) could refine survival predictions for those receiving preoperative treatment [11]. With larger datasets, machine learning techniques could be employed to automatically discover complex interactions between variables; however, any such model should continue to be evaluated for fairness and interpretability. In fact, our study’s framework can serve as a template: future prognostic models in oncology should routinely include calibration checks (quantifying any over- or under-prediction) and bias audits across demographic and clinical subgroups. Researchers might explore more sophisticated fairness interventions—for instance, if a model is found to have higher false-positive rates in older patients, one could implement stratified prediction thresholds or reweighting methods to correct [33]. Moreover, extending fairness analysis to other relevant axes (such as socioeconomic status or hospital of treatment) would be valuable, especially in larger datasets where such information is recorded. We also encourage future work on defining acceptable fairness thresholds in the clinical context. Not all performance differences constitute actionable bias—small variations may be clinically inconsequential—so developing guidelines on what level of disparity warrants model adjustment will be important, possibly in conjunction with ethicists and regulators.

Finally, long-term follow-up and model updating should be considered. As TNBC treatments evolve (e.g., the introduction of immunotherapy or targeted agents), the prognostic factors and baseline hazard may change over time. Periodic retraining or recalibration of the model with new patient data will ensure it remains applicable to contemporary cohorts. This is an area for future work, potentially using online learning techniques or setting up a registry to collect outcomes prospectively. In summary, future research should validate our model in broader populations, enhance it with new data and methods, and continue the dual focus on accuracy and fairness that this study has exemplified.

### Conclusion

In conclusion, this study developed a prognostic Cox model for TNBC that is both predictively strong and fairness-aware. Using six easily obtained clinical predictors, the model achieved high discrimination (C-index ~0.8) and good calibration in internal validation, placing its performance on par with the best existing TNBC prediction tools. It confirmed that classical factors—notably the presence of metastasis at diagnosis, tumor grade, and extent of surgery (a proxy for tumor burden) —drive survival outcomes in TNBC, which is consistent with the broader literature on TNBC prognosis. Unlike most prior work, we also evaluated the model through the lens of algorithmic fairness, finding that its predictions are largely equitable across different patient groups, with no major biases detected. This represents a novel contribution, demonstrating that advanced prognostic models can be developed in line with ethical AI guidelines (ISO/IEC TR 24027:2021) to ensure no subgroup is unfairly advantaged or disadvantaged by the tool. Our study addresses an important gap by focusing on a MENA cohort, thereby providing region-specific insight and a model that may be better suited to Middle Eastern/North African patients than transposed Western models. The results have direct implications for improving TNBC risk stratification and personalized care in our region and offer a framework that can be emulated and built upon by researchers in other locales. Ultimately, the integration of fairness assessment in prognostic modeling, as exemplified here, will be crucial for fostering trust and transparency in clinical AI systems [32, 33]. By delivering a model that is clinically interpretable, prognostically useful, and aligned with fairness principles, we hope to contribute to more informed and equitable breast cancer care, supporting global efforts to reduce mortality and ensure that advances in predictive oncology benefit all patient groups.

## Abbreviations

AUC: area under the curve
AUROC: receiver operating characteristic area under the curve
BCSS: breast cancer specific survival
CI: confidence interval
C-index: concordance index
CoxPH: Cox proportional hazards
DFS: disease free survival
EFS: event free survival
ER: estrogen receptor
FPR: false positive rate
GBCI: Global Breast Cancer Initiative
HER2: human epidermal growth factor receptor 2
HRs: hazard ratios
IPCW: inverse probability of censoring weighted
MENA: Middle East and North Africa
OS: overall survival
pCR: pathological complete response
PH: proportional hazards
PR: progesterone receptor
ROC: receiver operating characteristic
TNBC: triple-negative breast cancer
TPR: true positive rate

## Declarations

### Availability of data and materials

The datasets analyzed during the current study are not publicly available due to ethical restrictions and institutional regulations imposed by the approving ethics committee.

### Copyright

© The Author(s) 2026.
